# Investigating the causes of casing deformation induced by faults and natural fractures in shale gas platform wells

**DOI:** 10.1371/journal.pone.0334353

**Published:** 2025-10-27

**Authors:** Juncheng Zhang, Jun Li, Wei Lian, Dian Wang, Shaokun Guo, Haoyong Huang, Junfeng Li

**Affiliations:** 1 College of Petroleum Engineering, China University of Petroleum Beijing, Beijing, China; 2 College of Petroleum, China University of Petroleum Beijing at Karamay, Karamay, Xinjiang, China; 3 Hainan Institute of China University of Petroleum Beijing, Sanya, Hainan, China; 4 PetroChina Southwest Oil and Gas Field Shale Gas Research Institute, Chengdu, Sichuan, China; Dawood University of Engineering and Technology, PAKISTAN

## Abstract

During the development of deep shale gas in Luzhou, southern Sichuan, faults and natural fractures caused extensive casing shear deformation, including those that happen during hydraulic fracturing and those that occur prior to fracturing. To investigate the mechanism of complex deformation of platform well casings caused by faults and natural fractures, this paper analyzed the characteristics of casing deformation and identified the primary types of deformation. By integrating microseismic signal data, it was determined that fault slip is the direct cause of casing deformation. Based on these findings, the Mohr–Coulomb criterion was used to evaluate fault slip conditions, using critical pore pressure as a threshold. A finite element model of platform well fracturing was built with actual engineering parameters. Simulating the fracturing process showed how pore pressure changed under different fracture conditions. Comparing these results with the critical pore pressure clarified how fractures at different scales impact casing deformation in platform wells. The findings suggest that: (1) Casing deformation in the Luzhou Block mainly involves shear deformation, with fault or large-scale fracture slip being the direct cause of these shear deformations; (2) Fault slip at the well location caused casing deformation during fracturing, while fluid migration along faults caused fault instability and slip near non-fractured wells, leading to casing deformation before fracturing; (3) If fractures of a scale similar to the well spacing are present within the platform area, nearby wells may also deform before fracturing. These results provide a scientific basis for understanding casing deformation mechanisms in shale gas platform wells and for developing effective prevention and control measures in the Luzhou Block.

## Introduction

The Sichuan Basin commenced shale gas exploration and development earlier, establishing China’s first shale gas field with production of “trillion cubic meters” and reserves of “hundred billion cubic meters”, ranking it as one of the most promising shale oil and gas basins in China [1,2]. By the end of 2023, a total of 2.98 × 10^12^ m^3^ of proven reserves had been declared in the Sichuan Basin, with an annual output of approximately 240 × 10^8^ m^3^, making it the leading area for shale gas development in China [3,4]. As development progresses, the exploitation of shale gas in the Sichuan Basin has gradually shifted to deep shale gas [5]. Taking the southern Sichuan region as an example, the deep shale gas resources in this area account for over 80% of the total shale gas resources in southern Sichuan [1]. The Luzhou Block is a key area for increasing the production of deep shale gas in southern Sichuan [6]. Shale gas development is technically challenging, requiring the application of horizontal well drilling and large-scale multi-stage hydraulic fracturing technologies. During the multi-stage fracturing process, the casing is subjected to a complex environment characterized by frequent variations in temperature and pressure, which frequently leads to casing deformation issues [7–10]. Casing deformation can result in a range of problems, including reduced fracturing stages, decreased construction efficiency, and inadequate reservoir stimulation, all of which significantly hinder the efficient development of shale gas reservoirs [11,12].

The reasons for casing deformation in shale gas wells are complex. Domestic and foreign scholars have conducted research on this issue from multiple aspects. Sugden et al. [13] found that during the fracturing process, the build-up section with poor cementing quality is affected by temperature changes, which weakens the compressive strength of the casing and leads to casing deformation. Lian et al. [14] believed that the redistribution of in-situ stress during the fracturing process causes uneven stress on the casing, resulting in deformation. Jiang et al. [15] discovered that when the casing is eccentric or the cement sheath is missing, multi-stage fracturing can cause stress concentration on the casing, thereby increasing stress and potentially leading to yield deformation. Tian et al. [16] believe that factors such as temperature effect, pressure effect, and bending effect during the fracturing process of shale gas wells can all affect the strength of the casing, resulting in deformation. Liu et al. [17] argue that after the fracturing fluid enters the microannulus between the casing and the cement sheath, the local load becomes excessive under the alternating action of temperature and pressure, which can lead to casing deformation. Xi et al. [18] suggest that lithologic interfaces, volume shrinkage of bound water in the annulus, and the activation of weak planes are the primary reasons for casing deformation. Benedetto et al. [19] based on the construction conditions of Vaca Muerta shale gas wells, found that the perforation section with poor cementing quality is prone to casing deformation. Through continuous research, Dong et al. [20], Chen et al. [21], Guo et al. [22], Zhang et al. [23], Zhang et al. [24], and Meng et al. [25] reached a consensus on the mechanism of casing deformation in shale gas wells through different approaches, including analyzing field data and geological characteristics, considering the relationship between focal mechanisms, and conducting numerical analysis using finite element models. They found that the fracturing fluid entering the formation causes changes in the pressure of the near-wellbore strata, which in turn disrupts the stress equilibrium on the fault or fracture surface. When the shear stress on the fault or fracture surface exceeds the shear strength, it leads to slip on the fault or fracture, which then shears the casing and causes deformation [26]. However, existing theories and understanding can only explain the casing deformation of wells that cross faults during fracturing, and cannot explain the casing deformation phenomenon of unfractured wells within the fracturing platform.

In the southern Sichuan region of the Sichuan Basin, particularly in the Luzhou Block, which serves as a representative area for deep shale gas development, complex geological structural features and well-developed fractures have led to severe casing shear deformation issues. This has resulted in widespread casing deformation both during and prior to hydraulic fracturing operations. As of May 2022, the Luzhou Block in southern Sichuan had 108 operational wells, among which 58 wells (53% of the total) exhibited casing deformation-28 wells showed deformation during fracturing, while 30 wells experienced deformation prior to fracturing. With the continuation of operations, the number of wells experiencing deformation both during and before fracturing has been growing.

The existing theoretical frameworks explaining casing deformation in shale gas wells cannot fully account for the casing deformation issues observed in the Luzhou Block. Therefore, an in-depth investigation into the causes of casing deformation prior to fracturing in platform wells within this block is necessary to clarify the underlying mechanisms. To identify the root cause of casing deformation in the Luzhou Block, this paper determined the direct cause of casing shear deformation in the Luzhou Block by analyzing field casing deformation patterns and integrating microseismic data. Then it applied the Mohr-Coulomb criterion to establish an expression for critical pore pressure. Taking into consideration the geological characteristics of the Luzhou Block, simplifies the microfracture, establishes the fracturing model of the platform wells under different fracture conditions, and simulates the change of pore pressure in the platform range under different natural fracture conditions. The change in pore pressure within the platform range is simulated, and the influence of platform well fracturing on the change in shale gas well casing within the platform range under different fracture conditions is analyzed. The findings provide valuable insights into the mechanisms behind both fracturing-induced and pre-fracturing casing deformation in the Luzhou Block. This research contributes to the prevention and control of casing deformation in the block, supporting the safe and efficient development of deep shale gas reservoirs in southern Sichuan.

### Background

#### Data analysis of casing damage and deformation of the Luzhou Block.

Based on the drilling and casing deformation statistics from July 2023, the casing damage and deformation conditions in well areas L03 and Y01 were statistically analyzed, with results presented in Table 1. The statistical results indicate that a total of 24 platforms exhibited significant casing damage and deformation, including 15 platforms in Well Area L03 and 9 platforms in Well Area Y01. Among these 24 platforms, 105 wells were affected by casing damage and deformation, with 53 wells located in area L03 and 52 wells in area Y01. All casing-damaged wells in the Well Area L03 exhibited casing deformation characteristics. Additionally, only Well L03H3 showed coupling damage, while Well L03H60-3 displayed corrosion damage. Similarly, all casing-damaged wells in the Well Area Y01 presented casing deformation features, with only Well Y01H2 demonstrating both coupling disengagement and corrosion damage. The casing damage situation in the Luzhou Block is predominantly characterized by casing deformation, indicating a particularly severe casing deformation.

**Table 1:** Statistics on casing damage and deformation in the Luzhou Block as of July 2023.

Well area	Platform	Well	Casing damage or deformation category
Well Area L03	L03H1	3	Casing deformation
	L03H3	3	Clamps damage, casing deformation
	L03H4	1, 3	Casing deformation
	L03H51	1, 2, 3, 4	Casing deformation
	L03H52	1, 2, 3, 4	Casing deformation
	L03H53	1, 2, 3, 4	Casing deformation
	L03H55	1, 2, 3, 4, 5, 6	Casing deformation
	L03H56	1, 2, 3, 4	Casing deformation
	L03H57	1, 2, 3, 4	Casing deformation
	L03H58	1, 2, 3, 4, 5, 6, 7	Casing deformation
	L03H59	1, 2, 3, 4	Casing deformation
	L03H60	3	Corrosion damage
		1, 2, 3, 4	Casing deformation
	L03H62	1, 2, 3, 4	Casing deformation
	L03H75	1, 2, 4	Casing deformation
	L03H79	4	Casing deformation
Well Area Y01	Y01H1	1, 3, 5, 6, 7, 8	Casing deformation
	Y01H2	1, 2, 3, 5, 6	Casing deformation
	Y01H3	1, 2, 3, 4, 5, 6, 7, 8	Casing deformation
		2	Clamp position disconnected, with obvious corrosion damage
	Y01H4	1, 2, 3, 4, 5, 6, 7, 8	Casing deformation
	Y01H40	1, 3, 4, 5, 6	Casing deformation
	Y01H10	1, 2, 3, 4	Casing deformation
	Y01H26	1, 2, 3, 4	Casing deformation
	Y01H29	1, 2, 3, 4	Casing deformation
	Y01H41	1, 2, 3, 4, 5, 6, 7, 8	Casing deformation

Luzhou Block uses CB/TP-140V grade casing with outer diameter 137.9 mm and inner diameter 114.3 mm. According to existing casing inner diameter logging data, L03 well area has 86 deformation points, with 52% having inner diameter ≤65 mm; Y01 well area has 62 deformation points, with 50% having inner diameter ≤65 mm. The degree of casing deformation in the Luzhou Block is significant.

**Fig 1:**
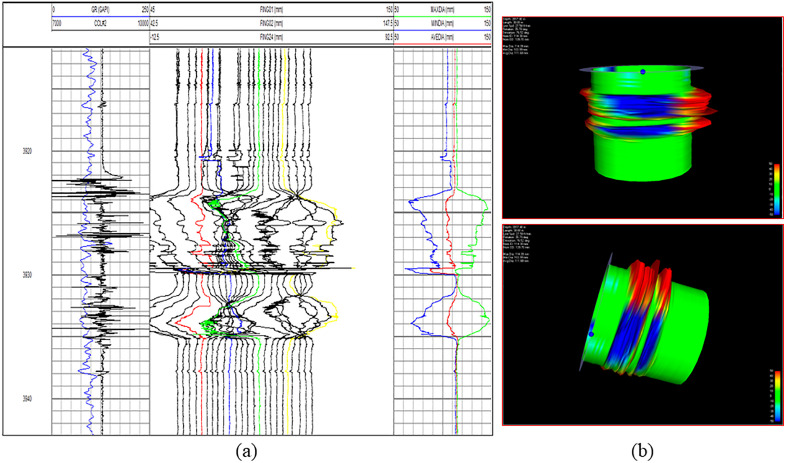
Logging information at the fifth level deformation point of Well L03H56-1. (a) Multi-arm caliper logging curves; (b) Three-dimensional imaging diagram of the deformation section casing.

The logging data of the MIT24 multi-arm caliper and the interpretation results of inner casing damage monitoring for 18 wells in Well Area L03 and 6 wells in Well Area Y01 indicate that the casing deformation degrees of different wells vary significantly, primarily at levels 3 and above, with a wide range of deformation. Moreover, the deformation degree of some wells is nearly 50%. For example, the minimum inner diameter of Well L03H56-1 in the well section of 3923.00–3935.60 m is 61.58 mm, with a deformation degree of 46.12%, belonging to level 5 deformation. Additionally, Well L03H1-3 has a minimum casing inner diameter of 58.52 mm with a deformation rate of 48.80%, while Well Y01H1-7 has a minimum casing inner diameter of 65.84 mm with a deformation rate of 42.40%. Among them, the multi-arm caliper measurement curve and 3D imaging results at the level 5 deformation point of Well L03H56-1 are shown in Fig 1. Judging from the above statistical data and logging results, the casing deformation in the Luzhou Block is severe, exhibiting obvious shear deformation characteristics.

#### Correlation analysis between casing deformation and fault slip in Luzhou Block.

**Fig 2:**
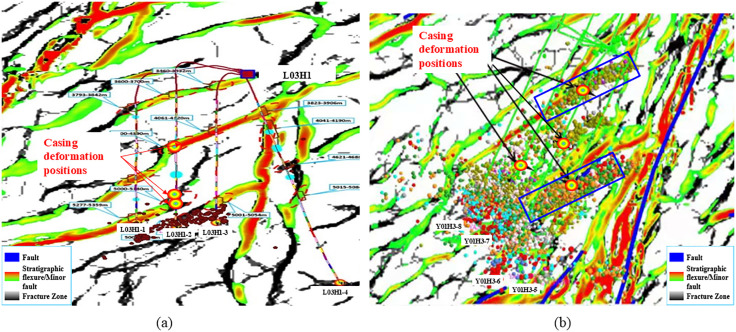
Platforms L03H1 and Y01H3 fault and part of the casing deformation location distribution. (a) Casing deformation locations and distribution of faults in well 2 of Platform L03H1 (b) Casing deformation locations and distribution of faults and microseismic signals in wells 5, 6 and 7 of Platform Y01H3.

The statistical results of the casing deformation points and fault distribution in the Luzhou Block indicate a high degree of coincidence between the casing deformation points and faults in this block. After the fracturing of wells 1, 2, and 4 on Platform H1 in the Well Area L03, multi-arm caliper logging revealed that well 3 deformed before fracturing. The casing deformation position coincides with the microseismic signal of well 2, as shown on the left side of Fig 2(a). Similarly, in the Well Area Y01, there is a significant correlation between the casing deformation points and the development positions of faults/fractures. Taking Platform H3 as an example, casing deformation occurred in wells 5, 6, and 7. Microseismic signals show that the natural fractures near the deformation points were activated, and the casing deformation positions highly coincide with the microseismic signals, as shown in Fig 2(b). Analysis of the microseismic signals and casing deformation positions reveals that fracturing can activate natural fractures or faults, resulting in casing deformation.

**Fig 3:**
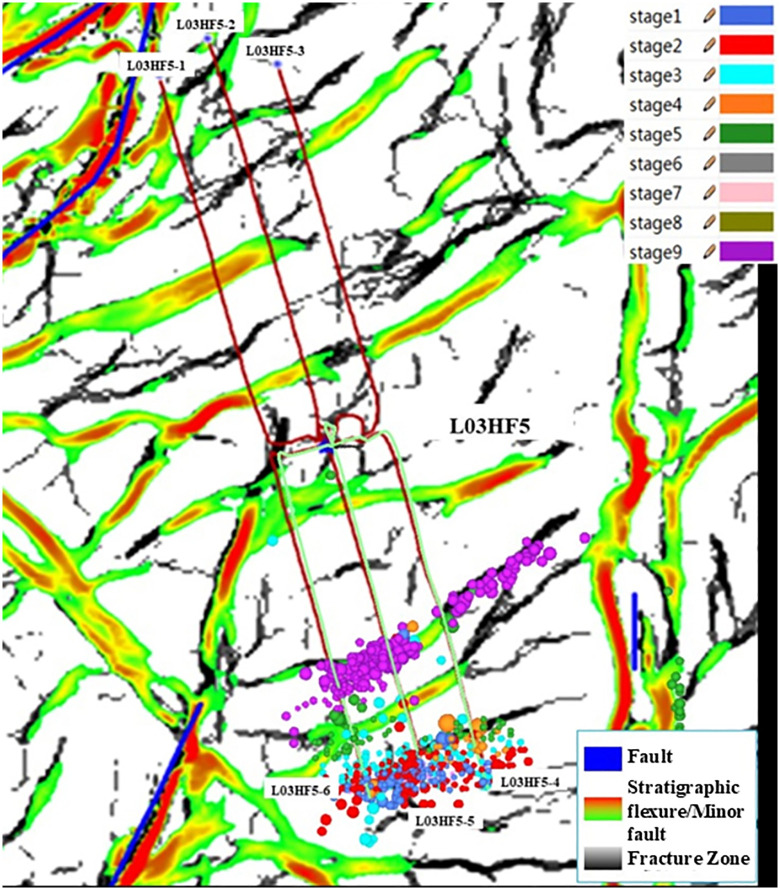
Distribution of Cumulative Microseismic Signals in Well 6 of Platform L03HF5.

Following the fracturing of wells 3 and 6 on the Platform L03HF5, obstructions occurred during the construction of wells 1, 2, 4, and 5. Inspection revealed that the casings of these four wells were deformed. Geological data indicate that small faults have developed in the toe areas of wells 1, 2, and 3 in the northern part of the platform, characterized by complex geological formations. Furthermore, the toe areas of wells 1, 2, and 3 are interconnected by fractures, and casing deformation has occurred. The other parts of the horizontal section of these three wells have no obvious faults and fractures, and no deformation has occurred. Wells 4, 5, and 6 in the southern part have large faults in the heel end and middle of the horizontal section. It can be clearly observed from the microseismic signal monitoring diagram of the 9th stage fracturing of Well HF5–6 (Fig 3) that the microseismic events penetrate wells 4, 5, and 6 and completely coincide with the fault zone. Therefore, it can be concluded that fracturing operations in well 6 induced activation of the fault where the well is located. The flow of fracturing fluid along the fault also caused fault activation at wells 4 and 5, resulting in casing deformation. The casing deformation locations at wells 1, 2, and 3 are concentrated in the toe area where small faults are well-developed, and the deformation causes should be identical to those at wells 4, 5, and 6. Furthermore, as no fracturing operations were conducted on adjacent platforms during this period, the casing deformation at the Platform HF5 is unrelated to neighboring platforms and should be attributed to fracturing operations within the platform itself that communicated with faults, causing deformation in either the subject well or adjacent wells.

Combining the casing deformation location, microseismic signals, and fault development data, it can be judged that the direct cause of casing shear deformation in the Luzhou Block is that the fracturing fluid flowed to the fault, which activated and slipped, leading to the casing deformation due to the shear effect.

## Materials and methods

Fracture fluid entering the fault and activating its slip is the direct cause of casing shear deformation, which can explain the casing shear deformation in fractured wells; however, the mechanism for deformation in adjacent unfractured wells still needs further clarification. For analysis, a finite element model is used to calculate pore pressures at different positions during multi-stage fracturing of platform wells, and a critical pore pressure calculation model (pore pressure at fault instability) is established based on the field-applied critical injection pressure (the difference between pore pressure at fault instability and initial pore pressure), to compare the calculation results of the two models for judging fault activation status.

### Finite element calculation model of pore pressure during multi-stage fracturing of platform wells

Faults and large-scale fractures have developed in the Luzhou Block, and most of the platform development wells pass through these faults, which can be easily activated when fracturing leads to an increase in pore pressure within them. To simulate the multi-stage fracturing process of platform wells in shale reservoirs, ABAQUS, a finite element commercial software, is used to obtain the variation pattern of pore pressure around the wells during the multi-stage fracturing process.

### Finite element model

**Fig 4:**
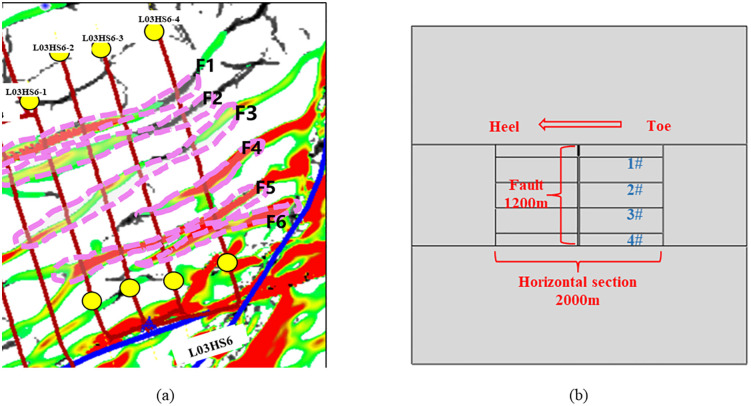
Finite element model reference and corresponding physical model. (a) Distribution of faults and well locations on the Platform L03HS6; (b) Physical model of platform wells containing only faults.

Due to the variety of fault and fracture development yields, the fault and fracture development of Platform L03HS6 (see Fig 4 (a)) is referred to facilitate the analysis. The micro-fractures in the formation are simplified, and the faults and different large-scale fractures are used as typical features for the study.

This paper refers to the field of fracturing design data and analyzes the scenario where faults and large-scale fractures are perpendicular to shale gas wells. A 2D numerical model is established for multi-stage fracturing of four wells on a single platform, considering faults and natural fractures of different scales. Given the presence of pore pressure, in-situ stress, and fracturing fluid pressure, the mesh type is set to pore-fluid/stress-coupled elements (CPE4P), and the model employs a structured tetrahedral mesh. In order to avoid the influence of boundary effects and simulate the real hydraulic fracturing at the same time, the model is set as a square with a side length of 4,000 m × 4,000 m. The length of the horizontal section of the four horizontal wells is unified at 2,000 m, which is located in the center of the model, and the spacing between the wells is 300 m. The half-length of fracturing stimulation for each well is 150 m. To investigate the influence of faults on horizontal wells within the platform during multi-well fracturing operations, a fault measuring 1,200 m in length (extending across all four horizontal wells) with a thickness of 20 m is modeled. The simulation setup includes three fracturing stages per well, with the nearest fracturing stage positioned 25 m from the fault. Two comparative models are additionally established: (1) Each well is subjected to an additional fracturing stage; (2) An extra fracturing stage is implemented while altering the fracturing sequence among wells. The fracturing model for the platform wells (fault-only case) is illustrated in Fig 4(b).

**Fig 5:**
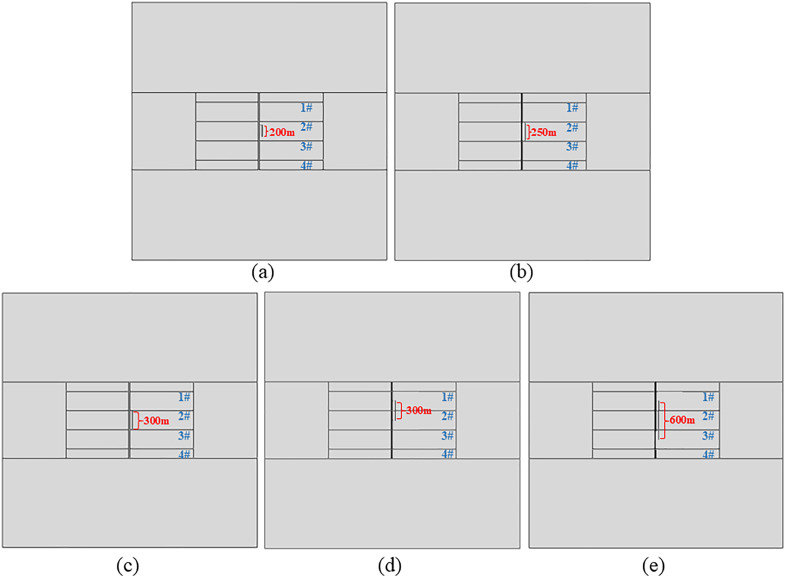
Platform wells model under different fracture conditions. (a) Fracture 200 m long between two wells; (b) Fracture 250 m long between two wells; (c) Fracture 300 m long between two wells; (d) Fracture length 300m through well 2; (e) Fracture length 600 m through wells 2 and 3.

Fractures are extremely developed in the target block, and in order to study the influence of different fracture conditions on the safety of platform wells on the premise that faults already exist in the platform range, both faults and large-scale fractures are considered on the basis of the fracturing model of platform wells. It is assumed that the fracture lengths are 200 m, 250 m, 300 m, and 600 m, which are divided into four categories: fracture whose length does not exceed the well spacing (300 m), fracture whose length reaches the well spacing and is connected to the platform well, fracture running through a single well, and fracture running through two wells. In the model of this paper, the fracture is taken to be 40m from the fault, and the same setting is set to fracture 3 stages for each well. The platform well model containing the fault and different scales of fracture is shown in Fig 5.

### Material parameters and boundary conditions of the model

The relevant basic parameters used in the model are shown in Table 2 below, where the parameters including the in-situ stress and initial pore pressure are obtained by taking the average value from the data of L03H1-3 well and the test results of rock mechanical parameters in the reservoir.

**Table 2:** Parameters of the finite element numerical model.

Model parameters	Value
Formation/fault rock elastic modulus (GPa)	40
Formation/fault rock Poisson’s ratio	0.23
Maximum horizontal stress (MPa)	100
Minimum horizontal stress (MPa)	90
Vertical Stress (MPa)	95
Initial pore pressure (MPa)	75
Bottomhole fracturing pressure (MPa)	140
Shut-in Pressure (MPa)	77
Formation initial porosity ratio	0.05
Fault zone porosity ratio	0.15
Formation permeability (mD)	1
Fault Zone permeability (mD)	500

Zero normal displacement constraints were applied in both x and y directions to prevent displacement and rotation of the model boundaries. The Predefined function was used to impose the in-situ stress, modeled pore ratio and initial pore pressure on the boundary. Each fracturing stage was set up with fracturing and soak stages. The initial analysis step utilized the Geostatic module to balance the modeled in-situ stress. The remaining analysis steps employed the Soil module to establish the process of in-well step-by-step fracturing and platform-by-well sequential fracturing. When fracturing is carried out, the fracturing stage is set as a high-pressure source that is being fractured, and when the first stage of fracturing is completed, the well is stewed, and the second stage of fracturing and stewing is begun..., and in the same way, the first well is fractured and the second, third, and fourth are fractured after the first well is fractured. The fracturing time for each stage is set to 10,800 s, and the stewing well time is 21,600 s.

### Critical pore pressure calculation model

**Fig 6:**
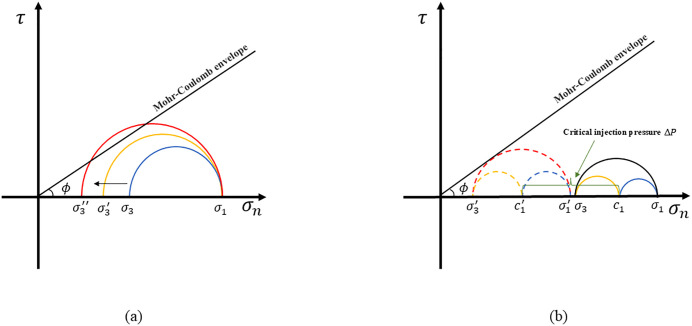
Mohr’s circle. (a) Mohr’s stress circle and Mohr-Coulomb envelope; (b) Critical injection pressure mechanism diagram.


The deep shale in southern Sichuan is primarily controlled by strike-slip faults, and the in-situ stress parameters are shown in Table 3. From the Well Area L03 to the Well Area Z03, the vertical stress gradually approaches or exceeds the maximum horizontal principal stress, and the minimum horizontal principal stress < vertical stress < maximum horizontal principal stress in the Luzhou Block, which is a distinctive feature of the stress state of strike-slip faults. The strike-slip faults are prone to slip from fault activation during fracturing, and the Mohr-Coulomb criterion (Eq. (1)) is generally used for fault slip risk assessment. And the Mohr’s Circle is shown in Fig 6.

**Table 3:** Statistics of in-situ stress parameters and fault type of typical wells in the deep layer of South Sichuan Province.

Block	Well area	Well	Depth (m)	In-situ stress (MPa)			Fault type
				Horizontal maximum	Horizontal minimum	Vertical	
Luzhou	L03	L03	3815.75	109.64	93.57	101.34	Strike-slip fault
		L03H57-3	3744.75	102.60	89.70	95.50	Strike-slip fault
		L03H79-4	3845.98	103.60	89.20	98.00	Strike-slip fault
	Y01	Y01H2-7	4146.67	115.26	99.09	105.31	Strike-slip fault
		Y01H10-3	3882.76	100.90	89.30	98.80	Strike-slip fault
Yuxi	Z03	Z01	4367.84	96.40	91.06	109.64	Normal fault

$$\left|\tau_{\text{0}}\right|=\mu \sigma_{n}+C$$ (1)

Where: $\tau_{\text{0}}$ is the shear stress at the fault surface, MPa; *µ* is the fault friction factor, dimensionless; $\sigma_{n}$ is the normal stress at the fault surface, MPa; *C* is the cohesion, MPa, when at a fault *C* = 0.

The Mohr’s stress circle is shown in Fig 6(a), which demonstrates that under identical fault orientations, greater principal stress differentials result in larger Mohr’s circles that approach the critical failure line more closely, indicating higher instability risks. Based on the computational relationships between in-situ stresses, normal stress, and pore pressure, the critical injection pressure at fault instability is determined, as illustrated in Fig 6(b). Under identical fault conditions, a higher pore pressure causes the Mohr’s circle to approach the critical failure line more closely, thereby increasing the risk of fault slip. During fracturing operations, the intrusion of fracturing fluid into the formation inevitably elevates the pore pressure in the vicinity of the wellbore. Consequently, the slip risk of faults surrounding the wellbore progressively intensifies as the fracturing operation continues. At the critical point of fault instability, the stress circle becomes tangent to the failure envelope, where the relationship between shear stress and effective normal stress is defined by Eq. (2):


$$\tau_{\text{0}}=\frac{\sigma_{n}^{\prime }-\sigma_{\text{3}}^{\prime }}{\text{2}}cos\phi$$ (2)

Where: $\tau_{\text{0}}$ is the shear stress when the fault is activated after fracturing, MPa; $\sigma_{\text{1}}^{\prime }$, $\sigma_{\text{3}}^{\prime }$ are the normal stress corresponding to the maximum and minimum principal stresses during fault activation, MPa; ϕ is the friction angle at the fault interface.

Based on Eq. 2 and Fig 5b, taking into account the relationship between principal stress, effective normal stress, and pore pressure, a mathematical transformation is performed to obtain Eq. (3):


$$\left\{ \begin{array}{c} \frac{\sigma_{\text{1}}^{\prime }-\sigma_{\text{3}}^{\prime }}{\text{2}}=\left(\frac{\sigma_{\text{1}}^{\prime }+\sigma_{\text{3}}^{\prime }}{\text{2}}\right)\text{sin}\phi \\ P_{p}^{\prime }=\frac{\sigma_{H\text{1}}+\sigma_{h\text{1}}}{\text{2}}-\frac{\sigma_{H\text{1}}-\sigma_{h\text{1}}}{\text{2}\text{sin}\phi }\\ P_{p}^{\prime }=\Delta P+P_{p} \end{array}\right.\;$$ (3)

Where: $P_{p}^{\prime }$ is the pore pressure when the fault is destabilized (critical pore pressure), MPa; $\sigma_{H\text{1}}$, $\sigma_{h\text{1}}$ for the maximum and minimum in-situ stress when the fault is destabilized, MPa; ΔP is the critical injection pressure when the fault is activated, MPa; $P_{p}$ is the original pore pressure at the fault, MPa.

During hydraulic fracturing, fracturing fluid is continuously injected into the formation, causing the pore pressure within the formation to increase continuously. According to the elastoplastic mechanics theory, the in-situ stress also increases synchronously. Therefore, the effective normal stress decreases as the pore pressure increases, and the critical pore pressure also decreases accordingly. This is consistent with the pattern reflected in Fig 6(b). For ease of analysis, the initial critical pore pressure is used for evaluation, that is, the critical pore pressure before fracturing is taken as the standard value to judge whether the fault will become unstable. The critical pore pressure at this time is given by Eq. (4).


$$P_{p\text{0}}^{\prime }=\frac{\sigma_{H}+\sigma_{h}}{\text{2}}-\frac{\sigma_{H}-\sigma_{h}}{\text{2}\mu \text{cos}\phi }$$ (4)

Where: $P_{p\text{0}}^{\prime }$ is the critical pore pressure at fault destabilization under initial conditions, MPa; $\sigma_{H}$, $\sigma_{h}$ is the initial maximum and minimum in-situ stress in the formation, MPa; μ=tanϕ, the Luzhou Block fault friction factor is 0.5-0.75 provided by the oilfield.

Eq. (4) indicates that when other conditions remain constant, the larger the friction factor, the higher the critical pore pressure, and the better the fault stability. Conversely, the greater the horizontal stress difference, the lower the critical pore pressure, and the worse the fault stability. When the pore pressure at the fault exceeds the critical value, it indicates fault instability. Calculated by the critical pore pressure model, combined with reservoir in-situ stress and a friction factor of 0.5, under the worst-case scenario, the calculated critical pore pressure of the fault is 83.82 MPa.

## Results and discussion

Based on the pore pressure at the fault/fracture obtained from the multi-stage fracturing simulation of platform wells, the critical pore pressure is compared to determine whether the fault or fracture will be activated. (Based on existing theories, this paper assumes that the activation of a fault will result in slip leading to casing deformation.) This method is used to analyze whether the fault or fracture will cause casing deformation without fracturing.

### Effect of large-scale faults on casing deformation

**Fig 7:**
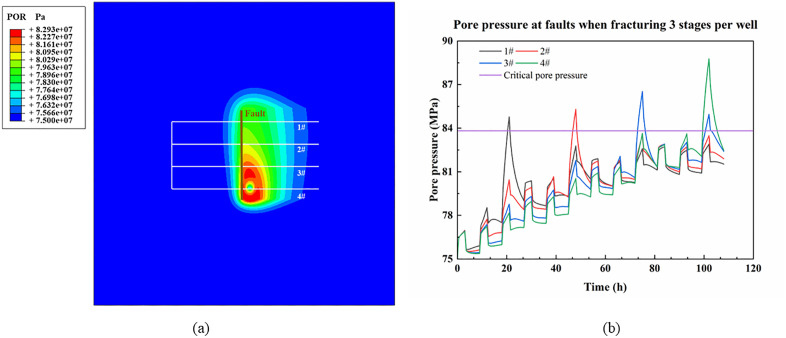
Pore pressure in the platform range when fracturing 3 stages per well. (a) Pore pressure cloud map; (b) Pore pressure variation pattern of platform wells at faults.

Fig 7(a) shows the pore pressure cloud maps during the staged and well-by-well fracturing of platform wells (with 3 fracturing stages per well) when only considering the fault. The variation patterns of pore pressure at the intersection points between different horizontal wells and the fault over the fracturing time are presented in Fig 7(b). The data for Fig 7(b) are detailed in S1 File.

From the pore pressure cloud diagram in Fig 7(a), it can be seen that the fracturing fluid is easy to seep along the faults with better permeability, and the fracture reformed area during fracturing. The high pressure after fracturing completion of platform wells is mainly concentrated in the fractured area between wells, with less influence on the unfractured area and the uncontrolled area of the platform. The color spectrum shows that the high-pressure transfer along the faults is obvious.

It can be seen from Fig 7(b): when the first fracturing stage is fractured, the nodes at the intersection of different wells and faults produce pressure changes, which rise from the initial pore pressure of 75 MPa in a short time, except that the change in pore pressure is small, less than 2 MPa. With the end of fracturing and entering the soak stage, the pressure decreases rapidly in all of them. However, due to high-pressure injection, the four wells retained some pressure increase after the end of the soak time. And the poor permeability of the formation leads to a relatively higher pressure the closer the fracturing stage is to the fault. It can be observed in the figure that the pressure at well 1 is slightly higher than that at well 2 after the completion of the first stage, followed by well 3 and minimized at well 4. It can be seen that as long as fracturing is carried out, it will affect the pore pressure of other wells on the platform, and the farther the distance, the smaller the effect.

With the fracturing, the pore pressure at the faults where the fractured wells are located increases rapidly. Under the current parameters, the fracturing location 25m from the faults leads to the pore pressure at the faults of the four wells exceeding the critical value, resulting in fault slip. This indicates that fracturing will lead to casing deformation in this well during fracturing, which is consistent with the results of field data analysis. Moreover, the pore pressure at the fault of the unfractured wells continues to increase with the increase in fracturing time, indicating that the higher the number of fractured wells on the platform, the higher the risk of fault destabilization at the remaining unfractured wells.

**Fig 8:**
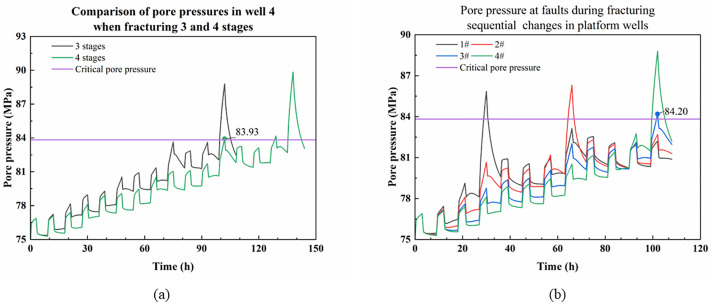
Variation pattern of pore pressure at faults under different conditions. (a) Comparison of pore pressure at the fault where well 4 is located when fracturing 3 and 4 stages per well; (b) Variation of pore pressure at the fault where the platform wells are located with fracturing of wells 1, 2 and 4.

When each well is fractured with 4 stages, the variation pattern of pore pressure at the intersection of horizontal wells and faults is similar to that when fracturing 3 stages, and the pore pressure at the faults grows more after the fracturing is completed, leading to activation of the faults in well 4 when fracturing well 3. Comparison of pore pressure at the fault of 4 wells under fracturing 3 and 4 stages presented in Fig 8(a). While changing the order of fracturing wells has a lesser influence on the results, the pattern remains unchanged. Therefore, one case of fracturing four stages in each well after changing the order is taken as an example for illustration, and for comparison, only the pore pressure at the completion of fracturing in three wells is shown in Fig 8(b). The S2 File contains the data used in Fig 8.

As shown in Fig 8, when each well is fractured in four stages, the pore pressure increase at the fault is more significant, and the peak pore pressure at the fault becomes higher as fracturing approaches the fault. This indicates that more fractured stages near the fault lead to greater pressure accumulation, making fault instability more likely. During the fracturing process, the pore pressure at the fault where the unfractured well is located keeps increasing due to pressure accumulation, which can easily lead to the pore pressure at the fault reaching a critical value when the well is not fractured. For example, well 4 in Fig 8(a), when fracturing to the 4th stage of well 3 (When each well is fractured for 3 stages, the time it takes for well 4 to be fractured to the third stage is the same as this), the pore pressure at the fault where well 4 is located reaches up to 83.93 MPa, which exceeds the critical pore pressure. At this time, well 4 was not fractured, but fault destabilization would still occur, which would lead to casing deformation at the fault without fracturing well 4.

Changing the fracturing sequence, fracturing wells 1-2-4-3 successively, the pore pressure at the fault of well 3 after fracturing wells 1, 2 and 4 under the current conditions will exceed the critical value, and destabilization occurs, as shown in Fig 8(b), the pore pressure at the fault of well 3 during the fracturing process of well 4 is the maximum of 84.20 MPa, which is consistent with the phenomenon of the Well L03H1-3 changing before fracturing. This is consistent with the phenomenon of casing deformation before fracturing in Well L03H1-3. It shows that regardless of the change of the fracturing sequence, the fault in the 4th well will be destabilized after fracturing in any 3 wells under the model condition, which leads to casing deformation in the 4th well without fracturing.

### Effect of fault and fracture characteristics on casing deformation

The Luzhou Block has extensively developed fractures, with faults and fractures of different scales often coexisting within the platform area. This study simplifies the geological conditions and categorizes them into four types for analysis.

(1) Fractures with lengths not exceeding well spacing (200 m and 250 m);
(2) Fractures reaching well spacing and connecting to platform wells (300 m);
(3) Fractures penetrating a single well (300 m);
(4) Fractures spanning two wells (600 m).

The objective is to investigate the combined effects of different fracture scales and fault interactions on casing deformation.

(1) Fractures with lengths not exceeding well spacing (200 m and 250 m)

**Fig 9:**
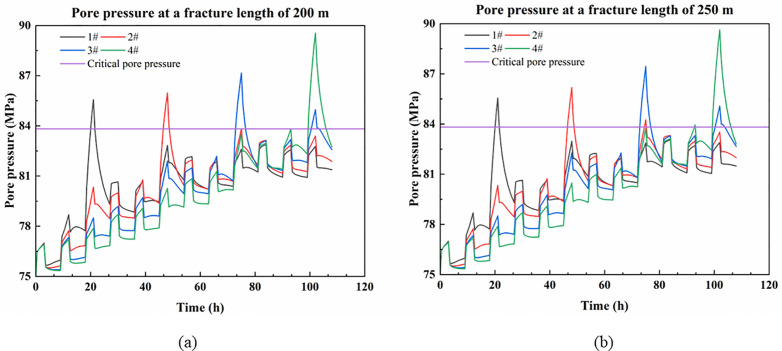
Pore pressure variation at the fault where the platform well is located when the fracture length does not exceed the well spacing. (a) Pore pressure at a fracture length of 200 m; (b) Pore pressure at a fracture length of 250 m.

The variation of pore pressure with fracturing time at the intersection of platform wells and faults for fracture lengths of 200 m and 250 m is shown in Fig 9. The data for Fig 9 are detailed in S3 File.

As shown in Fig 9, considering the inter-well fracture, the pore pressure at the fault under conditions of 200m and 250m fracture length increases overall compared to that without fracture. The longer the fracture, the higher the pore pressure, but the increase is smaller. Moreover, due to the limited fracture length, no connecting channel is established with the neighboring wells, resulting in a poor pressure transfer effect. Under the condition of fracturing only 3 stages in each well, it is impossible to activate the faults where the neighboring wells are located. Therefore, it can be seen that, namely, when the fracture length is smaller than the well spacing within the platform, the permeability of the formation within the platform is enhanced, but there is almost no significant change in the state of the platform wells.

(2) Fractures reaching well spacing and connecting to platform wells (300 m)

**Fig 10:**
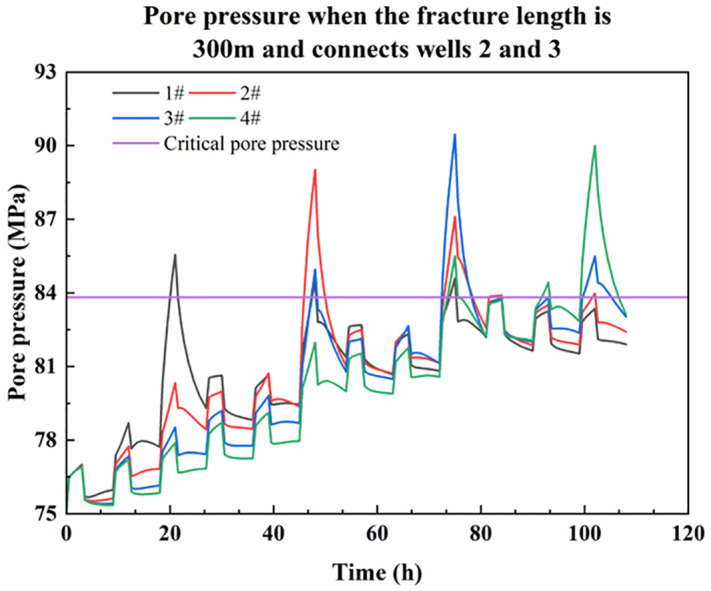
Pore pressure variation at the fault where the platform well is located when the fracture connects wells 2 and 3.

When the fracture length is 300m and communicates two adjacent wells, Fig 10 shows the variation of pore pressure at the intersection of the platform well and the fault with fracturing time. The S4 File contains the data used in Fig 10.

When the fracture length reaches the well spacing of 300 m and connects adjacent wells 2 and 3, there is no fracture shear wellbore because the fracture does not pass through wells 2 and 3. As shown in Fig 10, when fracturing reaches the fracture area of well 2, due to the existence of an advantageous channel for fracturing fluid flow between wells 2 and 3, the fracturing fluid will be connected to well 3 through the fracture, and the pore pressure around both wells 2 and 3 will increase significantly. At this time, the fault at well 3 is affected by the fracturing of well 2. The pore pressure increase is obvious and exceeds the critical value, activating the slip shear casing. Consequently, casing deformation will occur in well 3 without fracturing. If the deformation scale of well 3 is small enough to continue the operation, the peak pore pressure at the fault of well 3 will significantly exceed that caused by the fracturing of well 2 during continued fracturing, and a second set of changes may also occur. Due to the fracture connection, the permeability between wells 2 and 3 is enhanced. Consequently, the pore pressure at the fault where well 4 is located will also be significantly increased, and the continued fracturing of well 3 will lead to casing deformation before the fracturing of well 4.

(3) Fractures penetrating a single well (300 m)

**Fig 11:**
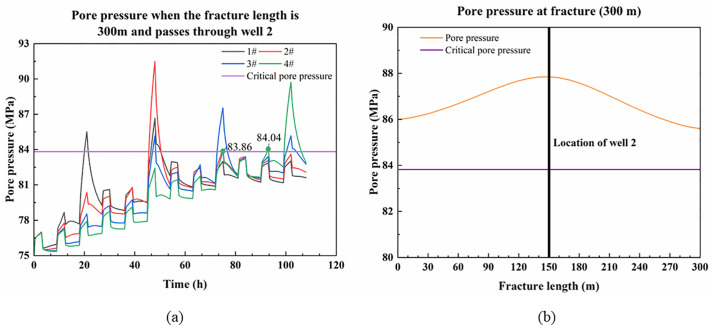
Pore pressure at the fault and the fracture when the fracture passes through well 2. (a) Variation in pore pressure at the fault where the platform well is located; (b) Pore pressure at fracture after fracturing stage 2nd of well 2.

When the fracture length is 300 m and runs through a single well, the variation of pore pressure at the intersection of the platform well and the fault with fracturing time is shown in Fig 11(a). Since the fracture extends through well 2 and there may be a case of fracture misalignment that shears the wellbore, the pore pressure at the fracture is illustrated in Fig 11(b) to show the situation when the pore pressure exceeds the critical value. The data for Fig 11 are detailed in S5 File.

As shown in Fig 11(a), when fractures penetrate well 2, fracturing operations cause a significant pressure rise around well 2. The high-pressure fluid rapidly propagates along fractures near the wellbore, affecting both wells 1 and 3, manifested as substantial pore pressure increases at their respective fault intersections. At this point, the pore pressure at the fault in well 3 exceeds the critical value, and fault activation will cause the casing in well 3 to deform before it is fractured. If well 3 continues fracturing, the peak pore pressure at its fault remains relatively low due to fracture conductivity. However, well 4 having accumulated excessive pressure during well 2’s earlier fracturing, which will cause the well 4’s casing to deform prematurely as the pore pressure exceeds a critical value when the well 3 is fractured near the fault.

Fig 11(b) demonstrates that, the pore pressure at the fracture exceeds the critical value during the 2nd stage fracturing of well 2. When such through-going fractures exhibit significant width and fault-like properties with potential displacement, this fracture will be prioritized for activation at well 2, causing casing shear deformation while preventing premature deformation at well 3. Subsequent fracturing of well 3 then benefits from pressure relief toward well 2 (which underwent one fewer fracturing stage), resulting in reduced pressure accumulation along the fault and avoiding premature deformation at well 4. At this point, well 2 is fracturing one less stage, and if well 3 continues to fracture, it will also vent pressure through the fracture to well 2. This leads to reduced pressure accumulation along the fault, thereby preventing premature casing deformation at well 4.

(4) Fractures spanning two wells (600 m)

**Fig 12:**
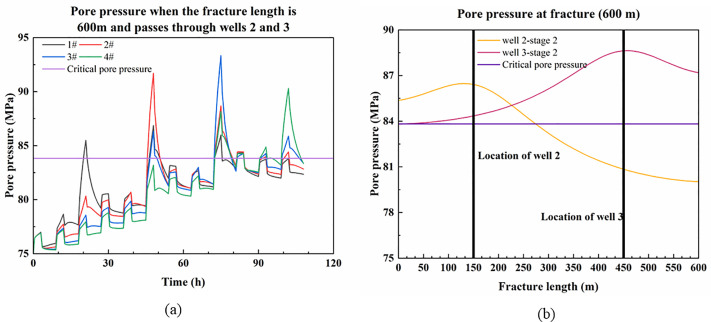
Pore pressure at the fault and the fracture when the fracture passes through wells 2 and 3. (a) Variation in pore pressure at the fault where the platform well is located; (b) Pore pressure at fractures after fracturing stage 2nd in wells 2 and 3.

When the fracture length is 600 m and runs through wells2 and 3, the variation of pore pressure at the intersection of platform wells and faults with fracturing time is shown in Fig 12(a). Since the fracture runs through wells 2 and 3, there may be a case of fracture misalignment that shears the wellbore. The pore pressure at the fracture is illustrated in Fig 12(b), where the pore pressure exceeds the critical value. The S6 File contains the data used in Fig 12.

As shown in Fig 12, when fractures penetrate both wells 2 and 3, the behavior is similar to the case where only well 2 is penetrated. If the penetrating fractures merely enhance permeability, fracturing well 2 only increases pore pressure at adjacent wells 1 and 3, causing premature casing deformation at well 3 before fracturing. In this scenario, if well 3’s casing deformation is minor, continued fracturing of well 3 may lead to secondary casing deformation at well 3 and premature deformation at well 4. However, if the penetrating fractures exhibit fault-like characteristics, fracture activation will occur preferentially during fracturing of the 2nd stage of the well 2 and the 2nd stage of the well 3, resulting in casing deformation during fracturing at wells 2 and 3. In this case, none of wells 2, 3, or 4 experience premature deformation.

### Casing shear deformation mechanism in Luzhou Block and engineering application

Based on simulation results and engineering phenomena observed in the Luzhou block, it is evident that the intersection of faults/fractures with the wellbore is a prerequisite. When localized activation of faults or fractures causes slip, it leads to shearing deformation of the casing in the intersecting area. Building upon this, and considering the specific geological characteristics of the site, the underlying causes can be categorized into three types:

Ⅰ. **Wellbore intersecting faults/large-scale fractures**

When a wellbore intersects a fault or large-scale fracture, multi-stage fracturing can induce instability in the fault or fracture, leading to casing deformation in the fractured well. Additionally, fracturing fluid may propagate along the fault. When the accumulated pressure exceeds the critical threshold, it can activate the fault near an unfractured well, resulting in shear-induced casing deformation.

Ⅱ. **Platform with major faults and inter-well fractures**

When a platform contains major faults with small-scale fractures between wells and fractures that can connect the wells, in addition to the mechanisms described in Category I, high-pressure fracturing fluid may propagate through these highly permeable fractures. This can lead to the accumulation of fault pressure exceeding critical thresholds in unfractured wells, resulting in casing deformation. The presence of inter-well fractures can make faults more prone to slip and casing more likely to deform during the fracturing process.

Ⅲ. **Platform with major faults and cross-well fractures**

When a platform encompasses both major faults and fractures intersecting platform wells, it integrates the mechanisms described in Categories I and II. Multi-stage fracturing of platform wells may induce slip along fractures intersecting the wellbores, causing casing deformation. Furthermore, fractures traversing the fracturing intervals of platform wells facilitate enhanced fluid flow, significantly increasing the risk of casing deformation in adjacent unfractured wells due to fracturing fluid flow.

The Luzhou Block is characterized by highly developed natural fractures with significant variations in scale and width, frequently exhibiting large-scale fractures. During hydraulic fracturing, frequent pressure communication phenomena occur, indicating the presence of extensive fractures connecting multiple wells. Combined with the results of this paper, it can be seen that the casing shear deformation in Luzhou Block is the result of the joint action of multiple mechanisms. Based on the geology of Luzhou Block, the type Ⅲ of casing deformation is most likely to occur, therefore, it is believed that the casing deformation of fractured wells caused by faults and fracture slips, and the casing deformation of unfractured wells caused by fracture penetration/connectivity to different wells are the main causes of casing shear deformation in Luzhou Block.

**Fig 13:**
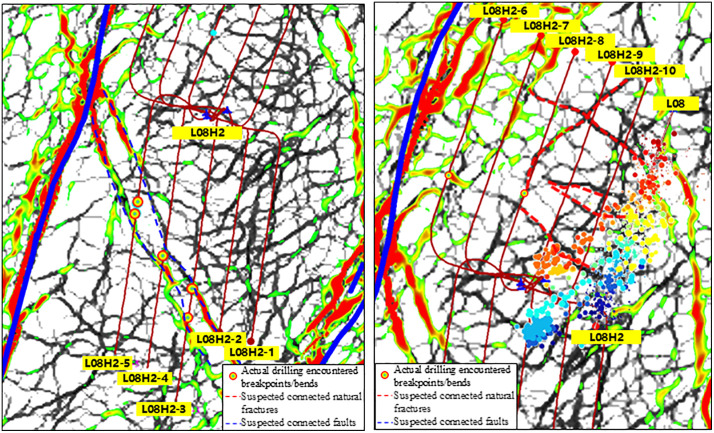
Overlay of multi-stage fracture planform distribution and fracture prediction with stageation categories in the Platform L08H2 region.

To address the causes of casing shear deformation in the Luzhou Block, when designing fracturing perforation parameters, attention should be paid to reasonably avoiding connected fractures and penetrating fractures, as well as preventing the formation of connected fractures between hydraulic fractures and natural fractures. Meanwhile, attention should be paid to controlling the fracturing scale to avoid cumulative multi-stage fracturing in front of faults and large-scale fractures. It is possible to consider increasing the avoidance perforation distance before and after faults or large-scale fractures, with only perforation but not fracturing performed in adjacent fracturing intervals to prevent casing shear deformation. Guided by this, the fracturing intervals of Platform L08H2 were designed as follows: areas passing through or adjacent to faults and large-scale fractures were defined as risk sections, where only perforation was performed without fracturing; the fracturing intervals adjacent to risk sections were defined as progressive sections, where the fracturing scale was controlled to avoid pressure accumulation. For the multi-stage fracture planar distribution and staged design of fracturing intervals of this platform, refer to Fig 13 and Table 4.

**Table 4:** Design parameters for fracturing stageation of each well on Platform L08H2.

Well location	Well number	Fracturing section length (m)	Total number of sections	Average section length (m)	Main sections			Progressive sections			Risk sections		
					Number of stages	Section length (m)	Percentage (%)	Number of stages	Section length (m)	Percentage (%)	Number of stages	Section length (m)	Percentage (%)
South	1	1703	24	71.0	22	70.0	91.7	2	82.0	8.3			
	2	1677	23	72.9	20	71.9	87.0	3	79.7	13.0			
	3	2400	32	75.0	22	73.0	68.8	6	75.0	18.8	4	83.8	12.5
	4	2272	31	73.6	24	72.0	77.4	3	73.0	9.7	4	80.3	12.9
	5	2146	30	71.5	19	68.8	63.3	5	72.0	16.7	6	79.8	20.0
North	6	1613	23	70.1	11	69.3	47.8	7	69.3	30.4	5	73.2	21.7
	7	1557	22	70.8	12	70.8	54.5	8	70.3	36.4	2	71.5	9.1
	8	1570	21	73.8	14	69.5	66.7	7	82.4	33.3			
	9	1602	22	72.8	16	71.3	72.7	6	77.0	27.3			
	10	1598	22	72.6	16	72.1	72.7	6	74.2	17.3			

The field implementation results show that the cumulative fracturing of Platform L08H2 exceeds 200 stages without casing shear deformation. It shows that the mechanism of casing shear deformation in Luzhou Block is reliable, and the design idea proposed accordingly can help prevent casing shear deformation in this block.

## Conclusions

The Luzhou Block is characterized by highly developed faults and fractures, with large-scale faults and fractures of varying scales generally present within each platform area. Based on the research conducted in this study, the following conclusions can be drawn:

(1) When fracturing is conducted near a fault, the fault or fracture where the fracturing well is located may be activated, leading to slip and casing shear deformation.
(2) When small-scale fractures exist between wells on a platform, the local formation permeability within the platform area increases, making it easier to activate faults during fracturing and causing casing deformation in the fracturing well. Additionally, continuous fracturing of multiple wells within the platform can lead to pressure accumulation at faults near unfractured wells, increasing the risk of fault activation and casing deformation.
(3) Fracturing will activate the fault where the fractured well is located when the fracture connects to a neighboring platform well. At the same time, the fracturing fluid will cascade to the neighboring well, increasing the pore pressure around the neighboring well. This can easily activate the fault where the neighboring well is located, resulting in casing deformation of the fractured well and casing deformation of the unfractured well that is connected to the fracture.
(4) When the fracture scale exceeds the well spacing and runs through multiple wells, fracturing construction will activate fractures and faults where the fractured wells are located, resulting in casing deformation of the fractured wells. Fracturing fluids will flow along the fracture, causing casing deformation in other unfractured wells that cross the fracture within the platform
(5) During fracturing operations, reasonable design of fracturing interval parameters—such as avoiding fracturing near faults and through-going fractures, preventing hydraulic fractures from connecting with natural fractures, and reducing the fracturing scale when approaching faults and large-scale fractures—can effectively prevent casing shear deformation.

## Supporting information

S1 File Pore pressure variation pattern of platform wells at faults when fracturing 3 stages per well. (XLSX)

S2 File Data that can be used to illustrate more fractured stages near the fault lead to greater pressure accumulation. (XLSX)

S3 File Pore pressure variation at the fault when the fracture length does not exceed the well spacing. (XLSX)

S4 File Pore pressure variation at the fault when the fracture connects wells 2 and 3. (XLSX)

S5 File Pore pressure at the fault and the fracture when the fracture passes through well 2. (XLSX)

S6 File Pore pressure at the fault and the fracture when the fracture passes through wells 2 and 3. (XLSX)
